# Multi-objective AGV scheduling in an FMS using a hybrid of genetic algorithm and particle swarm optimization

**DOI:** 10.1371/journal.pone.0169817

**Published:** 2017-03-06

**Authors:** Maryam Mousavi, Hwa Jen Yap, Siti Nurmaya Musa, Farzad Tahriri, Siti Zawiah Md Dawal

**Affiliations:** Centre for Product Design and Manufacturing, Department of Mechanical Engineering, Faculty of Engineering, University of Malaya, Kuala Lumpur, Malaysia; Beihang University, CHINA

## Abstract

Flexible manufacturing system (FMS) enhances the firm’s flexibility and responsiveness to the ever-changing customer demand by providing a fast product diversification capability. Performance of an FMS is highly dependent upon the accuracy of scheduling policy for the components of the system, such as automated guided vehicles (AGVs). An AGV as a mobile robot provides remarkable industrial capabilities for material and goods transportation within a manufacturing facility or a warehouse. Allocating AGVs to tasks, while considering the cost and time of operations, defines the AGV scheduling process. Multi-objective scheduling of AGVs, unlike single objective practices, is a complex and combinatorial process. In the main draw of the research, a mathematical model was developed and integrated with evolutionary algorithms (genetic algorithm (GA), particle swarm optimization (PSO), and hybrid GA-PSO) to optimize the task scheduling of AGVs with the objectives of minimizing makespan and number of AGVs while considering the AGVs’ battery charge. Assessment of the numerical examples’ scheduling before and after the optimization proved the applicability of all the three algorithms in decreasing the makespan and AGV numbers. The hybrid GA-PSO produced the optimum result and outperformed the other two algorithms, in which the mean of AGVs operation efficiency was found to be 69.4, 74, and 79.8 percent in PSO, GA, and hybrid GA-PSO, respectively. Evaluation and validation of the model was performed by simulation via Flexsim software.

## Introduction

### General

In today's competitive market, customer satisfaction plays a key role in companies’ market share. Therefore, organizations have shifted their strategy from manufacturing large quantities of a single product to a range of products, and improving the quality and on-time delivery. To meet these challenges, organizations should have a flexible manufacturing platform. In automated manufacturing environments, FMS provides wide flexibility and concurrent production of a wide variety of parts in small capacities. It comprises material handling devices like robots and AGVs, automated storage and retrieval systems (AS/RS) and workstations. AGVs are extensively used in FMSs because of their flexible structure and high compatibility [1, 2].

AGVs are driverless mobile vehicles that are computer-controlled (usually battery operated) and equipped with different guidance systems (optical, magnetic, laser, etc.) for automated functionality. They are categorized into two groups of load towing and load carrying (forked, mandrel, unit load deck, etc.) [3]. AGVs are well-suited for long-distance horizontal movement of materials from/to multiple destinations. They are also apt for repetitive/predictable material transportations and/or dangerous tasks [4, 5]. AGVs control system can be incorporated into the computer control of the production and storage equipment; thus, all the shop floor operations would be controlled using a computer system.

FMS performance increases by better coordination and scheduling of its components like AGV [6–8]. The term ‘scheduling’ refers to the process of allocating AGVs to tasks, taking into account the cost and required time for the operations to be done [9]. Efficient scheduling therefore would increase the productivity and reduce the delivery cost whilst the entire fleet is optimally utilized [7]. Although the AGV scheduling context has been studied before, given the diversity in objectives, limitations and considerations in scheduling problems, it is still an open area of research to improve it for real environment results. Improvement in the performance of an FMS can be expected by efficiently utilizing its resources and properly integrating and synchronizing their scheduling [9–11]. Literature has shown tendency toward multi-task scheduling of AGV systems and FMSs, in which the makespan minimization criterion is accompanied with several other criteria to entertain an actual-practice scheduling [12–14]. Heuristic techniques and evolutionary algorithms (EA) are the common optimization methods used to solve a multi-task scheduling problem [3, 15–17]. Some of the distinct researches in scheduling context that can assist new researchers in finding a pertinent literature to their specific objectives and methodologies are [18–21].

In the majority of the earlier works, makespan minimization was the main objective in scheduling practices as it keeps the resources utilization rate at a balanced level and ensures proper utilization of expensive FMSs [10, 22, 23]. However, those studies have discounted the importance of equal utilization of all the resources. Allocating a large number of AGVs may lead to a shorter makespan but it escalates the idle time of AGVs and costs [24]. AGVs are such expensive devices that determining the type and the appropriate number of them in an FMS can positively influence the profitability of the business [14, 17, 25–27]. Another issue in AGV scheduling is the charge of an AGV’s battery, where many studies do not consider the AGV’s battery charge and that leads to unrealistic scheduling models. Battery management in an AGV System (AGVS) is crucial as it can reduce the costs and increase the efficiency of the system [28, 29]. To address the above concerns, this research aims to schedule AGVs in an FMS environment by developing a multi-objective mathematical model that minimizes the makespan and number of AGVs while considering the AGV's battery charge. The model will be optimized using three evolutionary algorithms (genetic algorithm (GA), particle swarm optimization (PSO), and hybrid GA-PSO) and validated through simulation in Flexsim software.

## Problem descriptions and assumptions

The type and operation of FMS vary according to the different configurations being used. Therefore, the system configuration has to be established precisely prior to the scheduling of AGVs. The system configuration, assumptions and objective criteria in this study are presented in the following sections.

### Model derivation

This section explains the mathematical model development for AGV scheduling using the three criteria selected based on the literature review. The three criteria are categorized into two main objectives: (1) minimizing the makespan and (2) minimizing the number of AGVs while considering the AGV’s battery charge. Each sub-section explains the mathematical definitions used to develop the model. However, prior to the model development it is necessary to define the conditions and limitations considered in the model framework. Thus, the following conditions were defined for the model development:

- All AGVs have unit-load capacity.
- AGVs and machines operate continuously without breakdown.
- There are no traffic problems, collision, deadlock or conflict.
- AGV loading and unloading times are fixed and considered in travel times.
- AGVs can always park at their pick-up/drop-off (P/D) locations.
- The velocity of the vehicles is constant and vehicles move forward only.
- Loading/unloading (L/U) equipment such as pallets are sufficiently allocated as well as input and output buffer for machines.
- The machine-to-machine distance and L/U point-to-machine distances are known.
- Each machine operates only one product at a time.
- The setup times are included in the time of production.
- The AGVs are stored in the home until dispatching commands are allocated.

#### Notations

*n*: Total number of jobs
*j*, *j*′: Indexes of jobs, genes', and dimensions' code, *j*, *j*′ = 1, 2, …, *n*
*m*_*j*_: Total number of operations for each job *j*
*i*, *i*′: Indexes of operations, *i*, *i*′ = 1, 2, …, *m*_*j*, *j*′_
*θ*: Total number of operations for all of jobs
*z*: Number of AGVs
*a*, *a*′: Index of AGVs, *a*, *a*′ = 1, …, *z*
*y*: Index of new AGV
*J*_*j*_: Job number *j*
*O*_*ji*_: Operation *i* from job *j*
*M*_*ji*_: Assigned machine for *O*_*ji*_
*p*_*ji*_: Processing time of *O*_*ji*_
$p_{ji}^{s}$
Start time of processing *O*_*ji*_
$p_{ji}^{e}$
End time of processing *O*_*ji*_
*H*: Load/unloading point (Home)
*A*^*a*^: AGV number *a*
*T*_*ji*_: Related task to *O*_*ji*_ (Moving from *M*_*j*(*i*−1)_ to *M*_*ji*_ or *H* to *M*_*ji*_)
$T_{ji}^{a}$
Assigned *A*^*a*^ to do task *T*_*ji*_
*T*^*a*^: A collection of operations that have done by *A*^*a*^
*T*: A collection of *T*^*a*^
*MS*: Makespan
*PS*: Population size for GA
*r*: Index of chromosomes, *r* = 1, …, *PS*
*e*: Index of genes, e = 1, …, *θ*
*C*_*r*_: Chromosome
*G*_*e*_: Gene
*CR*: Crossover rate
*Pm*: Mutation rate
*G*_max_: Maximum gene code
*Iter*_max_: The maximum iterations
*Iter*: The current iteration number
*t*: Iteration number
*S*^*t*^: Swarm size at iteration *(t)*
*α*: Index of particles, *α* = 1, …, *S*^*t*^
*PR*_*α*_: Particle
*d*: Dimension, *d* = 1, …, *θ*
*ω*: Inertia factor
*ω*_max_: Maximum inertia factor
*ω*_min_: Minimum inertia factor
$v_{\alpha d}^{t}$
The velocity of *α*^*th*^ particle on *d*^*th*^ dimension at iteration *(t)*
$v_{\alpha d}^{t+1}$
The velocity of *α*^*th*^ particle on *d*^*th*^ dimension at iteration *(t+1)*
$V_{\alpha }^{t}$
The velocity of *α*^*th*^ particle in the swarm at iteration *(t)*
$q_{\alpha d}^{t}$
The position of *α*^*th*^ particle on *d*^*th*^ dimension at iteration *(t)*
$q_{\alpha d}^{t+1}$
The position of *α*^*th*^ particle on *d*^*th*^ dimension at iteration *(t+1)*
$Q_{\alpha }^{t}$
The position of *α*^*th*^ particle in the swarm at iteration *(t)*
$B_{\alpha d}^{t}$
The best position of *α*^*th*^ particle on *d*^*th*^ dimension found so far
$G_{d}^{t}$
The global best position of the swarm on *d*^*th*^ dimension found so far
*φ*_1_ and *φ*_2_: Uniformly distributed random numbers in the interval [0, 1]
*C*_1_: Self-confidence
*C*_2_: Swarm confidence
*NA*: Number of AGVs to do all the operations
*CTO*_*ji*_: The time that operation *O*_*ji*_ completes
*CChA*^*a*^: Current battery charge of *A*^*a*^
$ChHT_{ji}^{a}$
Charge that *A*^*a*^ needed for doing the task *T*_*ji*_ and back home
$ChT_{ji}^{a}$
The battery charge that *A*^*a*^ consumes for doing *T*_*ji*_
*ChA*^*a*^: The total battery charge that *A*^*a*^ consumes for all of its operations
*CA*^*a*^: Current position of *A*^*a*^,(Can be *H*, *M*_*ji*_, *M*_*j*′*i*′_, and *M*_*j*(*i*−1)_)
*tCA*^*a*^: Time of current position of *A*^*a*^
$tT_{ji}^{a}H$
Time that *A*^*a*^ arrives home after doing *T*_*ji*_
$PT_{ji}^{a}$
Pick up point of *A*^*a*^ doing *T*_*ji*_, (*P* represents pick up point and can be *H*, *M*_*ji*_, *M*_*j*′*i*′_, and *M*_*j*(*i*−1)_)
$tPT_{ji}^{a}$
Pick up time of *A*^*a*^ doing *T*_*ji*_
$rPT_{ji}^{a}$
The time that *A*^*a*^ reaches pick up place of *T*_*ji*_
$DT_{ji}^{a}$
Drop off point of *A*^*a*^ doing *T*_*ji*_,(*D* represents drop off point and can be *H*, *M*_*ji*_, *M*_*j*′*i*′_, and *M*_*j*(*i*−1)_)
$tDT_{ji}^{a}$
Drop off time of *A*^*a*^ doing *T*_*ji*_
$rDT_{ji}^{a}$
The time that *A*^*a*^ reaches drop off place of *T*_*ji*_
*μ*: A large positive number
$tCPT_{ji}^{a}$
The travel time of *A*^*a*^ from its current point to reach the start point of *T*_*ji*_
*γ*: A coefficient for transforming energy consumption to time
$UT_{ji}^{a}$
Unloaded time of *A*^*a*^ doing *T*_*ji*_
*UtA*^*a*^: Total unloaded time of *A*^*a*^
*ItA*^*a*^: Total idle time of *A*^*a*^
$WT_{ji}^{a}$
Waiting time of *A*^*a*^ doing *T*_*ji*_
*WtA*^*a*^: Total waiting time of *A*^*a*^
$LT_{ji}^{a}$
loaded time of *A*^*a*^ doing *T*_*ji*_
*LtA*^*a*^: Total loaded time of *A*^*a*^
$RT_{ji}^{a}$
Running time (loaded + unloaded) of *A*^*a*^ doing *T*_*ji*_
*RtA*^*a*^: Total running time (loaded + unloaded) of *A*^*a*^
$\bar{TuCh}$
The time that AGV is being charged
*BU*: Battery usage percentage of *A*^*a*^
*AE*: Efficiency of AGV’s operation (%)
*λ*: A coefficient for determining when a new AGV should be added
*L*: Number of objectives
*β*: Index of *δ*, *β* = 1, …, *L*
*δ*: The *β*^*th*^ weight of the *β*^*th*^ objective function
*ψ*: A ratio to make balance among objectives with different ranges of value
*f*(*x*): Fitness function

#### Minimizing the makespan

This step involves calculating makespan (*MS*) which is the time required for all operations to be completed. Makespan can be expressed by

$$MS=\underset{}{max}\left\{\left(tDT_{ji}^{a}+p_{ji}\right)\right\}$$ (1)

$$tDT_{ji}^{a}=tCA_{}^{a}+UT_{ji}^{a}+WT_{ji}^{a}+LT_{ji}^{a}$$ (2)

Subject to:

$$\begin{array}{cc} CTO_{ji}\geq p_{ji}^{s} & \forall i=1 \end{array}$$ (3)

$$\begin{array}{cc} tPT_{ji}^{a}\geq 0 & \forall \;T_{ji}^{a}\in T^{a} \end{array}$$ (4)

$$\begin{array}{cc} p_{ji}^{s}\;\geq \;tPT_{j(i+1)}^{a}-p_{ji} & \forall \;j,i \end{array}$$ (5)

$$\begin{array}{cc} p_{ji}^{s}-p_{j\left(i-1\right)}^{s}\geq p_{ji}+LT_{ji}^{a} & \forall \;j,\;i=2,\ldots ,\;m_{j} \end{array}$$ (6)

$$\begin{array}{l} (p_{ji}^{s}-p_{j\prime i\prime }^{s}-p_{j\prime i\prime }+\mu \left|M_{ji}-M_{j\prime i\prime }\right|\geq 0)\;\vee \\ \;\left(p_{j\prime i\prime }^{s}-p_{ji}^{s}-p_{ji}+\mu \left|M_{ji}-M_{j\prime i\prime }\right|\geq 0\right)\;\forall \left(j,i,j\prime ,i\prime \right) \end{array}$$ (7)

$$\begin{array}{l} (tPT_{jm_{j}}^{a}-tPT_{j\prime i\prime }^{a}-LT_{j\prime i\prime }^{a}+\mu \left|T_{ji}^{a}-T_{j\prime i\prime }^{a}\right|\geq 0)\;\vee \\ \;\left(tPT_{j\prime i\prime }^{a}-tPT_{jm_{j}}^{a}-LT_{ji}^{a}+\mu \left|T_{ji}^{a}-T_{j\prime i\prime }^{a}\right|\geq 0\right)\;\forall \left(j,m_{j},j\prime ,i\prime \right) \end{array}$$ (8)

Constraint number 3 ensures the feasibility of completion time of the first operation of each job. Constraints number 4 and 5 ensure the feasibility of pick up time of operations. Inequality number 6 describes the operations precedency constraint. Inequalities number 7 and 8 represent the operation and the AGV un-overlapping constraints respectively.

#### Minimizing the number of AGVs

This step involves calculating the number of AGVs, which is denoted by *NA* by considering the AGVs battery charge sufficiency. Number of AGV can be expressed by

$$NA=\underset{}{\text{max}}\left\{a\right\}|\;T^{a}\in T$$ (9)

Subject to

$$A^{a}\text{ is assigned to }T_{ji}\;(\text{to create }T_{ji}^{a})\;if\left\{ \begin{array}{l} ChA^{a}\geq ChHT_{ji}^{a}\\ \;\wedge \;\\ \;\\ \left\{ \begin{array}{l} tCPT_{ji}^{a}<tCPT_{ji}^{a\prime }\;\\ \;\vee \\ \begin{array}{ccc} tCPT_{ji}^{a}=tCPT_{ji}^{a\prime } & \wedge & a<a\prime \end{array} \end{array}\right. \end{array}\right.$$ (10)

$$ChHT_{ji}^{a}=\gamma (UT_{ji}^{a}+\;LT_{ji}^{a}+(tT_{ji}^{a}H-\;tDT_{ji}^{a}))$$ (11)

$$\begin{array}{l} A^{y}|{}_{y\;is\;a\;new\;AGV}\text{ is assigned to }T_{ji}\text{ (to create }T_{ji}^{y}\text{)}\\ \;if\left\{tCPT_{ji}^{y}+RT_{ji}^{y}\leq \lambda \left(tCPT_{ji}^{a}+RT_{ji}^{a}\right)\right\} \end{array}$$ (12)

where Eq 10 makes sure the assigned AGV has enough battery charge to do the job and return home, while it chooses the AGV which takes less time to reach the point. Eq 11 calculates the charge that AGV needs to do the job and return home. As battery-run-time of an AGV and battery-charging-time can be defined depending on the type of batteries used, charging methods, charge rate, application, manufacturer, and assignments the vehicles perform, *γ* has been defined to adopt to any kind of battery, charging method, etc. The automatic and opportunity battery charging considered here, which on average, an AGV charges for 10–12 minutes every hour [30, 31]. Eq 12 determines the suitable time for adding a new AGV.

#### Multi-objective evaluation

Decision makers refer to choosing a solution out of all the efficient solutions as a posteriori approach. Pareto is one well-known pioneer in multi-objective optimization problems. In this method, Pareto-optimal set is a group of best trade-off schedules, and Pareto-front refers to a set of Pareto solutions [32]. Overall fitness function formulation for two objectives is described by

$$f\left(x\right)=\delta_{1}f_{1}\left(x\right)\text{+}\psi \text{(1-}\delta_{1}\text{) }f_{2}\left(x\right)$$ (13)

Where *δ*_1_ is the weight of first objective function and *ψ* is a ratio to make balance among objectives with different ranges of value [33–35], which is defined by

$$\psi =\frac{max\;f_{1}(x)}{max\;f_{2}(x)}$$ (14)

### AGV specifications

This step involves calculating specifications of AGV number *a* including its total running time denoted by *RtA*^*a*^ (loaded (*LtA*^*a*^) + unloaded time (*UtA*^*a*^)), its waiting time (*WtA*^*a*^), its idle time (*ItA*^*a*^), its consumed battery charge (*ChA*^*a*^), its battery usage percentage (*BU*), and its efficiency (*AE*) by Eqs 15 to 27.

$$\;UtA^{a}=\sum_{\begin{array}{l} \;j,i\\ T_{ji}^{a}\in T^{a} \end{array}}^{}UT_{ji}^{a}$$ (15)

$$UT_{ji}^{a}=tPT_{ji}^{a}-tCA^{a}=\{\begin{array}{lllll} tHT_{ji}^{a}\text{- }tM_{j(i-1)} & if & CA_{}^{a}=M_{j(i-1)} & \wedge & i=1\\ tHT_{ji}^{a}-tM_{ji} & if & CA_{}^{a}=M_{ji} & \wedge & i=1\\ tHT_{ji}^{a}-tM_{j\prime i\prime } & if & CA_{}^{a}=M_{j\prime i\prime } & \wedge & i=1\\ 0 & if & \{\begin{array}{l} CA_{}^{a}=H\\ \;\vee \\ CA_{}^{a}=M_{j(i-1)} \end{array} & \begin{array}{c} \wedge \\ \wedge \end{array} & \begin{array}{c} i=1\\ i\neq 1 \end{array}\\ tM_{j(i-1)}T_{ji}^{a}-tH & if & CA_{}^{a}=H & \wedge & i\neq 1\\ tM_{j(i-1)}T_{ji}^{a}-tM_{ji} & if & CA_{}^{a}=M_{ji} & \wedge & i\neq 1\\ tM_{j(i-1)}T_{ji}^{a}-tM_{j\prime i\prime } & if & CA_{}^{a}=M_{j\prime i\prime } & \wedge & i\neq 1 \end{array}$$ (16)

$$\;LtA^{a}=\sum_{\begin{array}{l} \;j,i\\ T_{ji}^{a}\in T^{a} \end{array}}^{}LT_{ji}^{a}$$ (17)

$$LT_{ji}^{a}=tDT_{ji}^{a}-tPT_{ji}^{a}=\{\begin{array}{lllll} tM_{ji}T_{ji}^{a}-tHT_{ji}^{a} & if & PT_{ji}^{a}=H & \wedge & i=1\\ tM_{ji}T_{ji}^{a}-tM_{j(i-1)}T_{ji}^{a} & if & PT_{ji}^{a}=M_{j(i-1)} & \wedge & i\neq 1 \end{array}$$ (18)

$$\;WtA^{a}=\sum_{\begin{array}{l} \;j,i\\ T_{ji}^{a}\in T^{a} \end{array}}^{}WT_{ji}^{a}$$ (19)

$$\begin{array}{ll} WT_{ji}^{a}= & \{\begin{array}{lllllll} p_{j(i-1)}^{e}-rPT_{ji}^{a} & if & rPT_{ji}^{a}<p_{j(i-1)}^{e} & \wedge & rDT_{ji}^{a}\geq p_{j\prime i\prime }^{e} & \wedge & i\neq 1\\ \left(p_{j(i-1)}^{e}-rPT_{ji}^{a}\right)+\left(p_{j\prime i\prime }^{e}-rDT_{ji}^{a}\right) & if & rPT_{ji}^{a}<p_{j(i-1)}^{e} & \wedge & rDT_{ji}^{a}<p_{j\prime i\prime }^{e} & \wedge & i\neq 1\\ p_{j\prime i\prime }^{e}-rDT_{ji}^{a} & if & \{\begin{array}{c} rDT_{ji}^{a}<p_{j\prime i\prime }^{e}\\ \vee \\ rDT_{ji}^{a}<p_{j\prime i\prime }^{e} \end{array} & \begin{array}{c} \wedge \\ \wedge \end{array} & \begin{array}{l} i=1\\ rPT_{ji}^{a}\geq p_{j(i-1)}^{e} \end{array} & \begin{array}{c} \wedge \end{array} & \begin{array}{c} i\neq 1 \end{array}\\ 0 & if & \{\begin{array}{c} rDT_{ji}^{a}\geq p_{j\prime i\prime }^{e}\\ \vee \\ rDT_{ji}^{a}\geq p_{j\prime i\prime }^{e} \end{array} & \begin{array}{c} \wedge \\ \wedge \end{array} & \begin{array}{l} i=1\\ rPT_{ji}^{a}\geq p_{j(i-1)}^{e} \end{array} & \begin{array}{c} \wedge \end{array} & \begin{array}{c} i\neq 1 \end{array} \end{array}\\ & \text{Subject to}\;M_{ji}≜\;M_{j\prime i\prime } \end{array}$$ (20)

$$ItA^{a}=MS-RtA^{a}-\bar{TuCh}$$ (21)

$$RtA^{a}=\sum_{\begin{array}{l} \;j,i\\ T_{ji}^{a}\in T^{a} \end{array}}^{}RT_{ji}^{a}$$ (22)

$$RT_{ji}^{a}=LT_{ji}^{a}+UT_{ji}^{a}$$ (23)

$$ChA^{a}=\sum_{\begin{array}{l} \;j,i\\ T_{ji}^{a}\in T^{a} \end{array}}^{}ChT_{ji}^{a}$$ (24)

$$ChT_{ji}^{a}=\gamma (LT_{ji}^{a}+UT_{ji}^{a})$$ (25)

$$AE=\frac{ChA^{a}}{\gamma ItA^{a}}\times 100$$ (26)

$$BU=\frac{ChA^{a}}{\gamma \left(MS-\bar{TuCh}\right)}\times 100$$ (27)

## Proposed algorithms

Three different evolutionary algorithms (EA) have been developed to optimize the mathematical AGV scheduling model. The three algorithms (GA, PSO, and hybrid GA-PSO) are later evaluated in terms of their strength and suitability for the scheduling problem.

### Genetic algorithm

GA is a search algorithm based on the mechanics of the natural selection process. The major steps of GA algorithm development according to the study objective are described in this section. However, readers for a thorough review of the GA are referred to publications of [36–40].

**Step 1. Initializing parameters.** It involves setting the parameters of the GA and creating the first generation of chromosomes based on the notations section. The general schematic for reading data for the problem is presented in Table 1. The first column shows a chromosome *(C*_*r*_*)* and the second one shows the genes (*G*_*e*_) of the chromosome. The encoding of each gene is presented in the third column, which will be discussed later.

**Table 1:** General schematic for reading data.

GA			PSO						
Chromosome *(C*_*r*_*)*	Gene number *(G*_*e*_*)*	Gene code *(j)*	Particle *(PR*_*α*_*)*	Dimension Number *(d)*	Dimension code *(j)*	Job *(J*_*j*_*)*	Operation *(O*_*ji*_*)*	Machine *(M*_*ji*_*)*	Processing time *(p*_*ji*_*)*
*C*_*1*_	*G*_*1*_	*1*	*PR*_*1*_	*1*	*1*	*J*_*1*_	*O*_*11*_	*M*_*11*_	*p*_*11*_
	*G*_*2*_	*1*		*2*	*1*		*O*_*12*_	*M*_*12*_	*p*_*12*_
	.	.		.	.		.	.	.
	.	.		.	.		.	.	.
	.	.		.	.		.	.	.
	*$G_{m_{1}}$*	*1*		*m*_*1*_	*1*		*$O_{1m_{1}}$*	*$M_{1m_{1}}$*	*$p_{1m_{1}}$*
	.	*2*		.	*2*	*J*_*2*_	*O*_*21*_	*M*_*21*_	*p*_*21*_
	.	.		.	.		.	.	.
	.	.		.	.		.	.	.
	.	.		.	.		.	.	.
	.	*2*		.	*2*		*$O_{2m_{2}}$*	*$M_{2m_{2}}$*	*$p_{2m_{2}}$*
	.	.		.	.	.	.	.	
	.	.		.	.	.	.	.	
	.	.		.	.	.	.	.	
		*n*		.	*n*	*J*_*n*_	*O*_*n1*_	*M*_*n1*_	*p*_*n1*_
	.	.		.	.		.	.	.
	.	.		.	.		.	.	.
	.	.		.	.		.	.	.
	*G*_*θ*_	*n*		*θ*	*n*		*$O_{nm_{n}}$*	*$M_{nm_{n}}$*	*$p_{nm_{n}}$*

**Step 2. Initializing population.** A set of chromosomes is needed to create a population. For constructing a chromosome, it is necessary to define a proper genetic representation (encoding) due to its significant effects on all the subsequent steps of the GA.

**Chromosome representation and encoding.** As it is shown in Table 1, each chromosome is formed by genes. The order of genes represents the priority of operations, which decreases from left to right; and the genes' code defines operations related to each job. Gene's code are the same as their job number so that all the genes related to *J*_*1*_ operations have the code ‘*1*’ and subsequently the code ‘*2*’ is given to all the genes related to the operations of *J*_*2*_, and so on. As the operations of each job are expected to be performed sequentially, the repetition of genes' code represents the corresponding operation number of the job as clearly described in the following example.

The number of genes in each chromosome equals the number of total operations in a job-set, which is expressed by:

$$\theta =\sum_{j,j\prime =1}^{j,j\prime =n}m_{j,j\prime }$$ (28)

**Chromosome generating.** A chromosome *(C*_*r*_*)* is a random construct of operations, which is expressed by

$$C_{r}=\left\{\left(m_{j,j\prime }\right)|j,j\prime =1,2,\ldots ,n\right\}=\left\{\underset{m_{1}}{\underset{︸}{\left(O_{11},O_{12},\ldots ,O_{1n}\right)}},\underset{m_{2}}{\underset{︸}{\left(O_{21},O_{22},\ldots ,O_{2n}\right)}},\ldots ,\underset{m_{j}}{\underset{︸}{\left(O_{n1},O_{n2},\ldots ,O_{nn}\right)}}\right\}$$ (29)

where *j*, *j'* are indexes of jobs, *j*, *j'* = *1*, *2*, *…*, *n* and *m*_*j*_,_*j'*_ = number of operations for each job. *O*_*ji*_ is the operation *i* of job *j*.

Chromosome coding and generating is explained below via an example of 3 jobs (*J*_*1*_, *J*_*2*_, *and J*_*3*_). Each job has 4, 3, and 5 operations respectively. Overall, there is *θ* = 4+3+5 = 12 operations. Therefore, the chromosome is a random construct of $\left[\underset{4}{\underset{︸}{1111}}\underset{3}{\underset{︸}{222}}\underset{5}{\underset{︸}{33333}}\right]$. A sample could be [221132313133]. Here, code ‘*1*’, ‘*2’*, and *‘3’* imply operations of *J*_*1*_, *J*_*2*_, and *J*_*3*_ respectively. From the left, the first *‘2’* represents the first operation of *J*_*2*_, the second ‘*2*’ represents the second operation of *J*_*2*_, the first *‘1’* represents the first operation of *J*_*1*_, and so on.

**Step 3. Multi-objective evaluation.** After initializing the population size, each chromosome is evaluated by minimizing the makespan and the AGV numbers, while considering the battery charge of AGV that are defined by Eqs 1 to 12. Then, the total fitness values of the efficient frontiers will be calculated based on Eq 13.

**Step 4. New population.** New population will be produced based on the below sub-steps: selection, crossover, elitism, and mutation operation.

**Selection.** To constantly enhance the population overall fitness, selection helps to discard the bad/weak designs and only keep the best ones in the population. It increases the likelihood of selection of fitter individuals for the next generation. There are a few different selection methods but their basis is the same. The tournament candidate selection, which is a proportionate random selection method, is used in this study. In this method, every individual in the population is paired at random with another. The fitness values of each pair is compared. The fitter individual of the pair moves on to the next round, while the other is disqualified. This continues until there are a number of winners equal to the desired number of parents. Then, this last group of winners is paired as the parents for new individuals [41].

**Fig 1:**
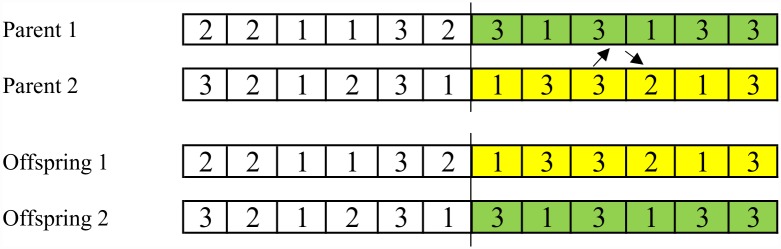
Example of one-point crossover.

**Crossover.** Crossover operator generates two new chromosomes for the next generation out of two selected chromosomes by exchanging some of their genes. This study employs two crossover operators based on partial strings exchange; a one-point crossover and a two-point crossover [42], where the one-point crossover is illustrated in Fig 1 based on the example in step 2.

**Fig 2:**
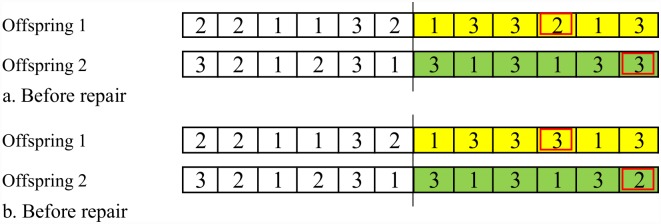
An example of repairing offsprings out of one-point crossover.

The offspring of crossover between the strings may not produce a legal encoding, for example, uncorrected number of operations per job may be seen. Therefore, they should be repaired and legalized. For repairing mechanism, counting from the left, the redundant genes will be deleted and compensate the missing ones, in order for each offspring to comprise all the operations of all the jobs. Repair mechanism is shown in Fig 2.

Legal chromosomes for the example in step 2 should include four code ‘*1*’, three code ‘*2*’, and five code ‘*3*’. In Fig 2a, counting from the left, in offspring 1, code ‘*2*’ is repeated four times, but there are three operations for *J*_*2*_, so it should repeat three times. There is one code ‘*2*’ that is redundant and should be replaced by the missing code. Code ‘*1*’ is repeated four times, which is correct, but code ‘*3*’ is repeated only four times, which should be 5 times. So in Fig 2b, the 4^th^ code ‘*2*’ will be replaced by number ‘*3*’. In offspring 2, number ‘*2*’ is repeated two times and number ‘*3*’ is repeated six times, so the last code ‘*3*’ will be changed to code ‘*2*’.

The number of crossovers is calculated based on the crossover rate (*CR*) and population size (*PS*) using

$$Number\;of\;crossovers=\frac{\left(CR\times PS\right)}{2}$$ (30)

**Mutation.** Mutation is another important operator of GA that initiates extra variability in a population to create and maintain the diversity. Mutation is not applied on chromosomes that are immune. The number of mutations in each generation is calculated using Eq (31) based on the mutation rate (*Pm*), population size (*PS*), and maximum gene code (*G*_max_).

$$Number\;of\;mutations≅\left(PS\times G_{\text{max}}\right)\times Pm$$ (31)

**Fig 3:**
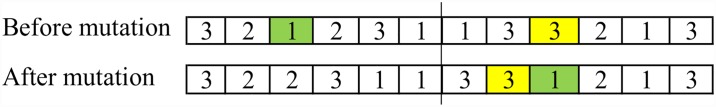
Example of shift mutation operator.

Shift mutation is used in this study [43] and it is shown in Fig 3. Based on the coding used in this study, chromosomes produced out of shift mutation are legal and no need to be repaired.

**Elitism.** The first three best chromosomes from each generation are transferred directly to the next generation in the elitism step to avoid annihilation. It is possible to maintain a fixed fitness value in some generations, but elitism makes sure they will never deteriorate.

**Step 5. Termination.** The loop of chromosome generation is terminated when the number of generation reaches its maximum, then the elite chromosome returns as the best solution.

### Particle swarm optimization

PSO is a population based stochastic technique inspired by social behaviour of bird flocking or fish schooling. Extensive reviews on PSO algorithm development can be found in [44–46]. PSO with limited information has been studied to avoid the waste of information [47]. Whereby population topologies and their performance had be studied [48]. Gao & collaborators proposed a method called Selectively-informed PSO (SIPSO) to allow the particles to learn at difference strategies based on their connections [49]. The PSO configuration for the mathematical model is described in details in the following steps:

**Step 1. Initializing parameters.** Initialization involves setting the parameters of the PSO and creating a group of particles to make the initial swarm. The general scheme for reading the data in the problem is presented in Table 1. The third column shows a particle (*PR*_*α*_) and the forth one shows dimensions of the particle *(d)*. The dimensions' codes are presented in the third column, which will be discussed later.

**Step 2. Initializing population (swarm).** A group of particles are needed to create a swarm. Each particle has position *(Q)* and velocity *(V)* in the search space at iteration *(t)*, where they are described briefly in the following sub-steps:

**Particle position.** First position of particle is filled by two digit numbers for *‘d’* dimensions of the particle using Eq 32. The number of dimensions is equal to the total number of operations, which is calculated by Eq 28.

$$q_{\alpha d}^{0}=\;q_{\text{min}}+\;\left(q_{\text{max}}-\;q_{\text{min}}\right)\times \varphi_{1}\;$$ (32)

where *q*_min_ = 0, *q*_max_ = 10 and *φ*_1_ is a uniform random number between 0 and 1.

**Particle velocity.** Initial velocities for the PSO particles are generated by the formula below:

$$v_{\alpha d}^{0}=\;v_{\text{min}}+\;\left(v_{\text{max}}-\;v_{\text{min}}\right)\times \varphi_{2}\;$$ (33)

where *v*_*min*_ = 0, *v*_max_ = 10 and *φ*_2_ is a uniform random number between 0 and 1.

**Step 3. Particle representation and encoding.** Every possible sequence of operations is considered as a particle, where the dimension of the particle represents each operation. Three sub-steps for encoding a particle are as follows: applying smallest position value (SPV) rule, assigning the dimensions' codes to the particles, and identifying sequence of operations in each job.

1. **Applying smallest position value (SPV) rule.** SPV is a rule that facilitates transformation of the continuous PSO algorithm to discrete cases applicable to all types of the scheduling problem [50]. As an example for better understanding of SPV rule, the corresponding sequence of a given continuous position like [0.3, 1.2, 0.9, 2.4] would be [4, 2, 3, 1]. In a descending order, ‘*0*.*3*’ is the smallest value and its sequence will be ‘*4*’; ‘*2*.*4*’ is the largest so its order in the group will be ‘*1*’.

**Assigning the dimensions' codes to the particles.** In this stage, the dimension’s codes as it is shown in the 6^th^ column of Table 1 are assigned to the particles. Dimension’s codes are based on the job number.

**Identifying sequence of operations in each job.** From the left side, the first appearance of a job number is assumed the first operation of that job (i.e., *O*_*j1*_). Similarly, the second time repetition of the same job number stands for the second operation of the same job (i.e., *O*_*j2*_) and so on. Once the first encountered generated number is assigned to the first operation of a job, this technique automatically handles the precedence constraints.

The stages of encoding an example with 3 jobs are shown in Table 2. Each job has 4, 3, and 5 operations respectively. The total operations are 12, which means the particle sample will have 12 dimensions being randomly generated using Eq 32 and shown in the first row of Table 2. In the second row of the Table, based on SPV rule, the numbers of 1 to 12 are assigned to the particles in an ascending order. In the third row, the dimensions’ codes based on the job numbers are given to the particles as follows: first four numbers are assigned to the first job, so their code is ‘*1*’, followed by the second three numbers assigned to the second job, so their code is ‘*2*’ and the remaining five numbers are assigned to the third job and their code is ‘*3*’. The sequence of operations in each job is shown in the fourth row of the Table 3. From the left, the first particle has the code ‘*1*’, so it belongs to job 1 and it is the first code ‘*1*’, which makes it the first operation of job 1 denoted by *O*_*11*_; the next code is ‘*2*’, so it belongs to job 2, but as it is the first code ‘*2*’, it is the first operation of job 2. The same structure is followed for the remained 10 operations.

**Table 2:** Encoding of a sample particle.

Particle sample	0.2	0.37	0.17	0.51	0.73	0.42	0.93	0.35	0.69	0.84	0.65	0.05
Applying SPV rule (giving the numbers from one based on ascending order)	3	5	2	7	10	6	12	4	9	11	8	1
Assigning the dimensions' codes to the particles	1	2	1	2	3	2	3	1	3	3	3	1
Identifying sequence of operations in each job	O_11_	O_21_	O_12_	O_22_	O_31_	O_23_	O_32_	O_13_	O_33_	O_34_	O_35_	O_14_

**Table 3:** AGV travel time among L/U point and machines for example 1.

	L/U	M_1_	M_2_	M_3_	M_4_	M_5_	M_6_
L/U	0	5	7	10	12	17	19
M_1_	15	0	2	5	7	12	14
M_2_	13	18	0	3	5	10	12
M_3_	10	15	17	0	2	7	9
M_4_	8	13	15	18	0	5	7
M_5_	3	8	10	13	15	0	2
M_6_	1	6	8	11	13	18	0

**Step 4. Multi-objective evaluation.** Once the swarm is generated, each particle is evaluated by minimizing the makespan and AGV numbers, while considering the AGV battery charge, which are defined by Eqs 1 to 12. Then, the total fitness values of the efficient frontiers will be calculated based on Eq 13.

**Personal best.** $B_{\alpha }^{t}$ represents the best position associated with the best permutation and fitness value of the particle *α* obtained so far and is called the personal best. For each particle, $B_{\alpha }^{t}$ can be determined and updated at each iteration.

**Global best.** *G*^*t*^ denotes the best position of the globally best particle achieved so far in the whole swarm.

**Step 5. New swarm.** To produce a new swarm, the position and velocity of the particles should be updated. Updated particles will be evaluated again according to the step four and the best local and global particle will be determined. This procedure will be repeated up to a point where the termination criterion is satisfied. The updating procedure is explained as follows:

1. **Updating the velocity of each particle.** The velocity of each particle is updated using
  $$v_{\alpha d}^{t+1}=\omega \;v_{\alpha d}^{t}+\;C_{1}\varphi_{1}(B_{\alpha d}^{t}-\;q_{\alpha d}^{t})\;+\;C_{2}\varphi_{2}(G_{d}^{t}-\;q_{\alpha d}^{t})$$ (34)
  where *C*_*1*_ is self-confidence while *C*_*2*_ is swarm confidence (common values of *C*_*1*_ and *C*_*2*_ varies between 0.1 and 0.5 but the values between 0.1 and 1 has been tested as well. In some literature, the value of 2 have also been observed). Inertia weight (*ω*) is a parameter to control the impact of the previous velocity on the current velocity [51, 52]. Let *ω* be varying with time by the following linear decreasing function.
  $$\omega =\omega_{\text{max}}-Iter\times \frac{\omega_{\text{max}}-\omega_{\text{min}}}{Iter_{\text{max}}}$$ (35)
2. **Updating the position of each particle.** The position of particle is updated using the updated velocity as below:
  $$q_{\alpha d}^{t+1}\;=\;q_{\alpha d}^{t}+\;v_{\alpha d}^{t+1}$$ (36)

**Step 6. Termination.** The loop of swarm groups is terminated when it reaches the maximum number of iteration, then the particle with global best returns as the best solution.

### Hybrid GA and PSO

**Fig 4:**
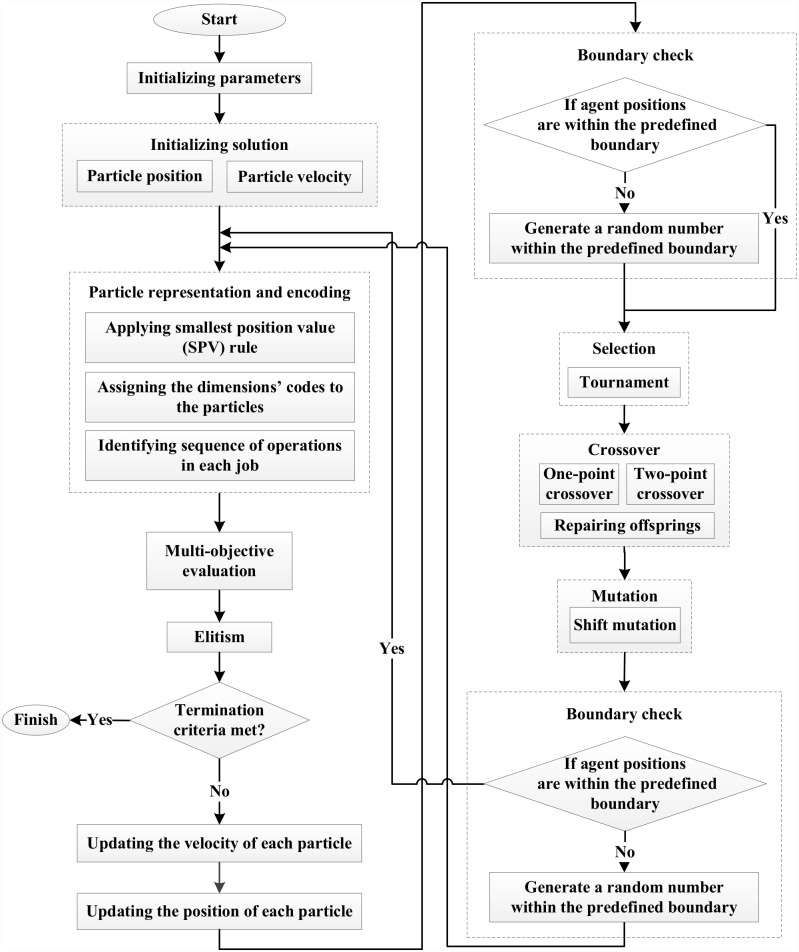
Flowchart of hybrid GA-PSO.

The PSO algorithm is a more robust optimization algorithms compared to many other algorithms as it can work almost independent from the problem. It does not require extensive prior-knowledge regarding the problem except the fitness evaluation of each particle [53]. On the other hand, GA has the capability of simultaneous evaluation of many points in the search area, which increases the probability of finding the global solution of the problem. Hybridization of EAs has been studied in many researches [54–56]. Generally, PSO functions based on the social interaction knowledge and all the individual particles will be considered in each generation. Unlike PSO, fitter chromosomes will be chosen in GA and the weaker ones will fade away from generation to generation [57]. Hence, by integrating the advantages of the compensatory properties of PSO and GA, their hybrid is used to obtain better result [58–60]. In the proposed GA-PSO algorithm for this study, after generating and evaluating the initial swarm and after position and velocity updating, the crossover operation has been used in the GA segment to avoid premature convergence; and a mutation operation was applied to maintain the diversity of the swarms. Elitism step was also performed to improve immune particle filter. Fig 4 illustrates the steps of hybrid GA-PSO and some parts of programming codes are listed in S1 Appendix.

## Computational results and discussion

To validate the model, two numerical examples have been used. The first example had 6 jobs (*J*_*1*_, …, *J*_*6*_) processing on 6 machines (*M*_*1*_, …, *M*_*6*_), and each job with 2 to 5 operations. The second one with 15 jobs (*J*_*1*_, …, *J*_*15*_) processing on 10 machines (*M*_*1*_, …, *M*_*10*_), and each job with 1 to 5 operations [23, 61]. Tables 3 and 4 show the AGV travel time among L/U point and machines and Tables 5 and 6 demonstrate the processing time of every operation on the machines for both the examples.

**Table 4:** AGV travel time among L/U point and machines for example 2.

Min	L/U	M_1_	M_2_	M_3_	M_4_	M_5_	M_6_	M_7_	M_8_	M_9_	M_10_
L/U	0	8	12	18	13	12	22	9	21	20	25
M_1_	8	0	6	17	20	16	4	5	12	14	19
M_2_	12	6	0	19	8	7	9	17	24	13	21
M_3_	18	17	19	0	12	5	4	9	6	13	10
M_4_	13	20	8	12	0	10	15	12	21	4	18
M_5_	12	16	7	5	10	0	5	23	18	17	9
M_6_	22	4	9	4	15	5	0	2	12	15	17
M_7_	9	5	17	9	12	23	2	0	14	19	23
M_8_	21	12	24	6	21	18	12	14	0	11	18
M_9_	20	14	13	13	4	17	15	19	11	0	17
M_10_	25	19	21	10	18	9	17	23	18	17	0

**Table 5:** The processing time of every operation on the machines for example 1.

Job	1	1	1	1	2	2	2	3	3	3	3	3	4	4	4	5	5	6	6
Operation	1	2	3	4	1	2	3	1	2	3	4	5	1	2	3	1	2	1	2
Machine	M_2_	M_4_	M_5_	M_6_	M_3_	M_4_	M_6_	M_1_	M_2_	M_3_	M_1_	M_4_	M_6_	M_4_	M_5_	M_5_	M_1_	M_1_	M_2_
Operation time	30	21	24	27	15	24	13	16	21	18	14	25	25	19	20	33	21	27	31

**Table 6:** The processing time of every operation on the machines for example 2.

Job	1	1	1	1	1	2	2	2	3	3	4	4	4	4	5	6
Operation	1	2	3	4	5	1	2	3	1	2	1	2	3	4	1	1
Machine	M_7_	M_1_	M_9_	M_8_	M_3_	M_2_	M_10_	M_6_	M_5_	M_4_	M_1_	M_3_	M_5_	M_7_	M_10_	M_8_
Operation time	19	21	14	10	11	15	21	22	30	26	1	12	19	14	29	12
Job	6	6	7	7	8	8	8	8	8	9	9	9	10	10	10	10
Operation	2	3	1	2	1	2	3	4	5	1	2	3	1	2	3	4
Machine	M_6_	M_3_	M_2_	M_7_	M_9_	M_3_	M_4_	M_5_	M_8_	M_10_	M_9_	M_5_	M_5_	M_1_	M_8_	M_2_
Operation time	24	17	29	16	9	21	24	10	12	20	10	16	17	5	24	20
Job	11	11	11	11	12	13	13	14	14	14	14	14	15	15	15	
Operation	1	2	3	4	1	1	2	1	2	3	4	5	1	2	3	
Machine	M_7_	M_9_	M_8_	M_1_	M_6_	M_10_	M_1_	M_3_	M_4_	M_2_	M_10_	M_9_	M_4_	M_8_	M_7_	
Operation time	11	11	14	15	49	29	30	12	11	9	10	10	15	21	4	

**Fig 5:**
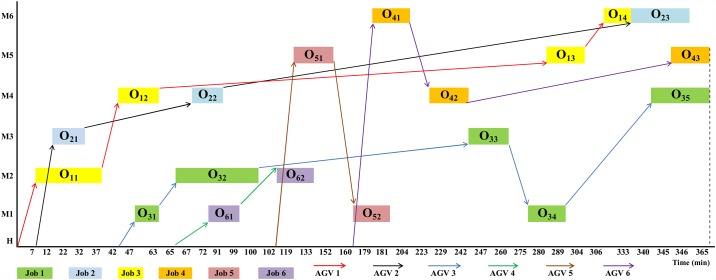
Gantt chart of a random sequence of the example using the six AGVs before optimization.

The makespan of scheduling before optimization by a random sequence and assigning one AGV to each of the six jobs for example 1 is shown in Fig 5. However, illustration of before optimization for example 2 was not possible due to its big figure size and detail.

Based on the experimental approach, the best setting of hybrid GA-PSO parameters was found to be the crossover and mutation rates of 0.2 and 0.08 respectively, *C*_*1*_ = 0.01, *C*_*2*_ = 0.9, *ω*_min_ = 0.01, and *ω*_max_ = 0.5. The algorithms were run 30 times, each run with a population size of 100 in 100 iterations, and their first two best results based on different AGV numbers are shown in Table 7.

**Table 7:** Test results of optimization algorithms (The first two best result of each) for both examples.

Algorithms		Example 1			Example 2		
		The first two best results		Mean computational time	The first two best results		Mean computational time
PSO	Fitness value	167.0877	172.5614	58.9164 Sec	625.9148	640.5532	261.0752 Sec
	Makespan	184	170		792	765	
	Number of AGV	3	4		6	8	
GA	Fitness value	163.0877	164.9474	60.3881 Sec	589.9148	590.234	269.0021 Sec
	Makespan	178	203		738	714	
	Number of AGV	3	2		6	7	
Hybrid GA-PSO	Fitness value	161.7544	162.2807	61.5003 Sec	566.2623	568.2339	273.7746 Sec
	Makespan	176	199		727	681	
	Number of AGV	3	2		5	7	

**Fig 6:**
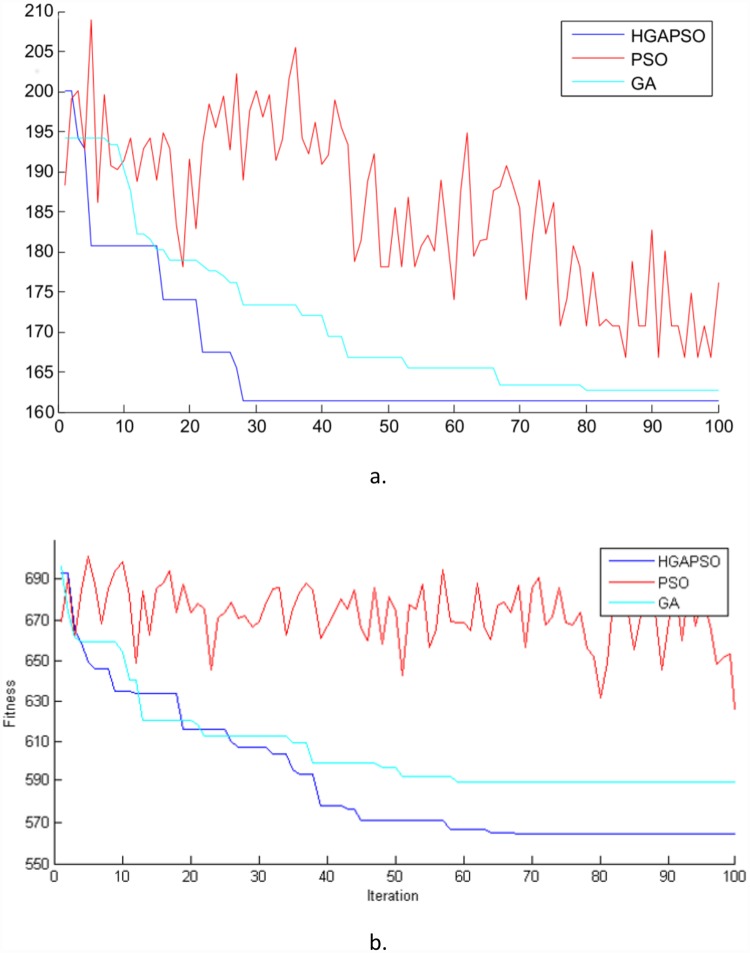
Performance of the different algorithms a. Example 1, b. Example 2.

The third column of Table 7 shows the best result of each algorithm, and the forth column shows the best result of each algorithm using a different number of AGVs compared with the first column. The fitness value in Table 7 has been calculated based on Eq 13, $\psi =\frac{max\;(MS)}{max\;(NA)}$, and $\delta_{1}=\frac{2}{3}$. *Max* (*MS*) was presumed to be equal to the sum of travel times and operation times. *Max* (*NA*) was presumed equal to the whole number of operations. All the steps were repeated for example 2. Fig 6 shows the performance of all the three algorithms at examples 1 and 2 based on the third and fifth column of the Table 7, respectively.

After the optimization, all the three algorithms were proved successful in decreasing the makespan and the required number of AGVs, and the optimized model using hybrid GA-PSO obtained the best result.

**Fig 7:**
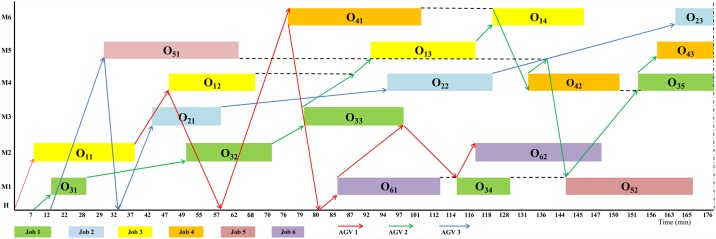
Gantt chart of the schedule of the example 1 after optimization by GA-PSO that employs three AGVs.

**Fig 8:**
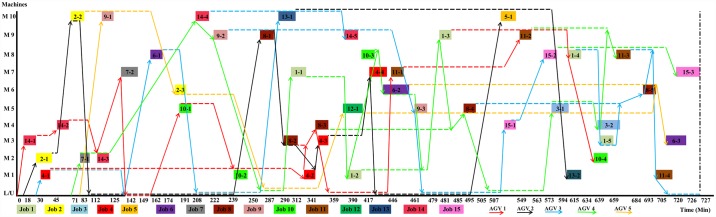
Gantt chart of the schedule of the example 2 after optimization by GA-PSO that employs five AGVs.

Fig 7 demonstrates the optimized sequence of Fig 5 using only 3 AGVs which is obtained by hybrid GA-PSO. In Figs 7 and 8, although the battery charge of AGV was considered, the path of AGVs going home for recharging is not shown to avoid extra complexity. Fig 8 shows the optimized sequence of example 2 obtained using hybrid GA-PSO (nearly half of the time points are not shown due to space limitations).

**Fig 9:**
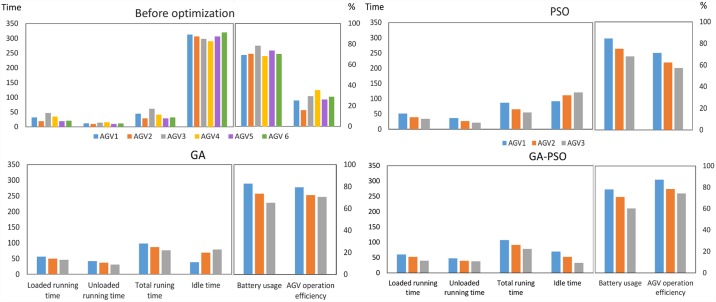
AGVs specification before and after optimization by PSO, GA, GA-PSO for the example 1.

To investigate the effect of optimization methods on AGV scheduling, AGVs' specification both before and after the optimization for example 1 were explored. The studied specifications are AGVs’ total running time (loaded and unloaded), idle time, battery usage, and operation efficiency computed using Eqs 15 to 27. In Fig 9, prior to the optimization, the AGVs total running time is low because a higher number of AGVs are employed with no intention to use their highest potential, compared to the optimized schedule, thus the idle time of AGVs has increased dramatically. The AGV number four (AGV_4_) had the highest operation efficiency (37.3%) before the optimization; although the scheduling model was designed to sequentially appoint tasks to AGVs based on their numbers’ order, so that AGV number one (AGV_1_) would have the highest operation efficiency level.

In GA-PSO, the makespan, number of AGVs and their idle time have been reduced, and consequently efficiency of AGVs' operation has enhanced (Fig 9). Potency of hybrid GA-PSO in solving scheduling problems and its superiority against its constituting algorithms have also been largely mentioned in other published studies [62–65]. Overall, application of the hybrid GA-PSO in scheduling studies is concluded to be more effective than its constituting EAs.

## Simulation by Flexsim

**Fig 10:**
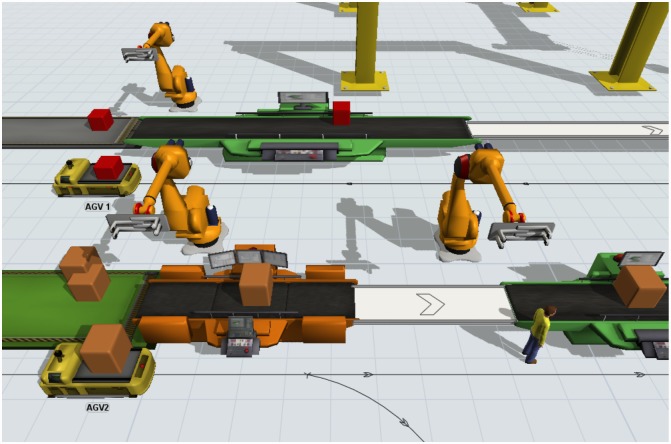
Simulation of the sample by Flexsim software.

In order to prove the feasibility of the proposed model, a simulation practice based on the above example has been performed using the Flexsim software. Fig 10 shows a scene from the simulation space. The simulation outcome confirmed the optimization results by obtaining equal makespan magnitude to all the three algorithms. The experimental results proved the validity and feasibility of the model, which provide useful reference for further research on the scheduling of AGV. Other experiments were also simulated to check the suitability and compatibility of the model to any kind of FMS configuration and environment. It can also be utilized for optimizing the objectives separately as well as in a combination.

## Conclusion

This research focused on the multi-objective AGV scheduling in an FMS using GA, PSO, and hybrid GA-PSO algorithms. A model was developed for the task scheduling of AGVs considering multiple objectives of minimizing makespan and number of AGVs, while considering the battery charge of AGVs. Using the numerical examples, near-optimum schedules for the combined objective functions were obtained. The inter-comparison of the three algorithms results showed that the hybrid GA-PSO yields the least makespan and AGV numbers. Literature has also largely exhibited the excellence of hybrid GA-PSO over its constituents in solving the scheduling problems [62–65], however the scheduling model proposed in this study distinguishes it from previous studies. The scheduling problem was further scrutinized by comparing the AGVs characteristics such as the total running time (loaded and unloaded), idle time, battery usage, and operation efficiency—before and after the optimization. It was found that after the optimization, despite a small rise in AGVs' total running time (loaded and unloaded), the AGVs' idle time reduction enhanced the AGVs' operation efficiency. This leads to effective utilization of AGVs and hence the overall efficiency of the system will be enhanced. In line with the experimental results, the AGV system simulation using the Flexsim software has also proved the feasibility of the developed model and suitability of the optimization algorithms for the scheduling problem. The developed model can be adopted to any FMS with different configuration and environment, and it can be applied for optimizing the objectives separately or in a combinatorial fashion. This research framework can be stretched out to examine newer algorithms and hybrids, and also employ more criteria in the scheduling model for further studies in this context.

## Supporting information

S1 Appendix Programming codes for hybrid GA-PSO. (PDF)
