# Supplementary material for: Multi-objective AGV scheduling in an FMS using a hybrid of genetic algorithm and particle swarm optimization
Source: PLoS One. 2017 Mar 6;12(3):e0169817. doi: 10.1371/journal.pone.0169817 (PMC5338791; doi:10.1371/journal.pone.0169817)
Supplement: S1 Appendix — (PDF) [file pone.0169817.s001.pdf]

Programming codes for Hybrid GA – PSO

```
tic;
%% Parameter tune
HGAPSO.Parameter.MaxGen = 100; %Generation, programmer
default=500
HGAPSO.Parameter.UB = 10;
HGAPSO.Parameter.LB = 0;
HGAPSO.Parameter.D = problem.Dimension;
HGAPSO.Parameter.N = 100; % population, programmer
default=20
HGAPSO.Parameter.C = [0.01, 0.9]; % [C1 , C2]
HGAPSO.Parameter.InertiaWeight = [0.01, 0.5]; % [Minimum inertia
weight, Maximum inertia weight,]
HGAPSO.Parameter.alfa = 0; % Parameter for linear
crossover, 0 by default
HGAPSO.Parameter.Pc = 0.9; % Crossover probability,
0.9 by default
HGAPSO.Parameter.Pm = 0.08; % Mutation probability,
0.1 by default
HGAPSO.Parameter.CrP = 0.2; % percentage iterations
with crossover
HGAPSO.Parameter.Elitism = [1, 0.0, 1.0]; % Elitism for GA starting
and ending percent from maximum iteration
% FOR Hybrid_GA_PSO
HGAPSO.Parameter.parallel = 1; % 1 = PSO output to GA, 0
= GA from old results
HGAPSO.Parameter.Reconstructor = 0.2; % the percentage which
worst results should be regenerated
[HGAPSO.output] = Hybrid_GA_PSO_1(HGAPSO.Parameter, problem);
HGAPSO.time = toc;
%% -----------------------Report Hybrid GA - PSO ---------------------------
HGAPSO.output.History.Y.Makespan(1,:) = [];
HGAPSO.output.History.Y.Eval(1,:) = [];
HGAPSO.output.History.Y.NAGV(1,:) = [];
% HGAPSO.output.History.X(1,:) = [];
%% ----------------------------- Save Iterations -----------------------------

Record(run).HGAPSO = HGAPSO;
Record(run).time = HGAPSO.time;
%% ----------------------------- Clear Variables -----------------------------
--
if problem.maxRun > 1
clear GA PSO HGAPSO
end
fprintf('Total Runing time for this Run: %4.4f Sec\n\n',Record(run).time);
fprintf('GA Runing time for this Run: %4.4f Sec\n\n',Record(run).GA.time);
fprintf('PSO Runing time for this Run: %4.4f Sec\n\n',Record(run).PSO.time
);
fprintf('HGAPSO Runing time for this Run: %4.4f
Sec\n\n',Record(run).HGAPSO.time);
end
% %% ----------------------------- Save Data ------------------------------
%
% filename = strcat('Record_', num2str(sum(clock)*1000), '.mat');
% save(filename,'Record');
%% ----------------------------- Reporting ------------------------------
```

```
Record = ReportRecord( Record );
% DrawGraph( Record, finalReport, maxRun);
% DrawGraohMakespan( Record, finalReport, problem);
% [Best.GA.Y,I] = min(Record.GA.output.History.Y.Eval);
% Best.GA.X = Record.GA.output.History.X(I,:);
% Best.GA.HistoryIndex = I;
%
% [Best.PSO.Y,I] = min(Record.PSO.output.History.Y.Eval);
% Best.PSO.X = Record.PSO.output.History.X(I,:);
% Best.PSO.HistoryIndex = I;
%
% [Best.HGAPSO.Y,I] = min(Record.GA.output.History.Y.Eval);
% Best.HGAPSO.X = Record.HGAPSO.output.History.X(I,:);
% Best.HGAPSO.HistoryIndex = I;
function Z = imlincomb(varargin)
%IMLINCOMB Linear combination of images.
% Z = IMLINCOMB(K1,A1,K2,A2, ..., Kn,An) computes K1*A1 + K2*A2 + ... +
% Kn*An. A1, A2, ..., An are real, non-sparse, numeric arrays with the
% same class and size, and K1, K2, ..., Kn are real double scalars. Z
% has the same size and class as A1 unless A1 is logical, in which case
% Z is double.
%
% Z = IMLINCOMB(K1,A1,K2,A2, ..., Kn,An,K) computes K1*A1 + K2*A2 +
% ... + Kn*An + K.
%
% Z = IMLINCOMB(..., OUTPUT_CLASS) lets you specify the class of Z.
% OUTPUT_CLASS is a string containing the name of a numeric class.
%
% Each element of the output, Z, is computed individually in
% double-precision floating point. When Z is an integer array, elements
% of Z that exceed the range of the integer type are truncated, and
% fractional values are rounded.
%
% Example 1
% ---------
% Scale an image by a factor of two.
%
% I = imread('cameraman.tif');
% J = imlincomb(2,I);
% figure, imshow(J)
%
% Example 2
% ---------
% Form a difference image with the zero value shifted to 128.
%
% I = imread('cameraman.tif');
% J = uint8(filter2(fspecial('gaussian'), I));
% K = imlincomb(1,I,-1,J,128); % K(r,c) = I(r,c) - J(r,c) + 128
% figure, imshow(K)
%
% Example 3
% ---------
% Add two images with a specified output class.
%
% I = imread('rice.png');
% J = imread('cameraman.tif');
% K = imlincomb(1,I,1,J,'uint16');
```

```
% figure, imshow(K,[])
%
% See also IMCOMPLEMENT.
% Copyright 1993-2013 The MathWorks, Inc.
% I/O spec
% ========
% A1, ... Real, numeric, full arrays
% Logical arrays also allowed, and are converted to uint8.
%
% K1, ... Real, double scalars
%
% OUTPUT_CLASS Case-insensitive nonambiguous abbreviation of one of
% these strings: uint8, uint16, uint32, int8, int16, int32,
% single, double
[ims, scalars, outputClass] = ParseInputs(varargin{:});
sameInputOutputClass = strcmp(class(ims{1}), outputClass);
if sameInputOutputClass
if imagePlusImage(ims,scalars)
Z = ims{1} + ims{2};
elseif image1MinusImage2(ims,scalars)
Z = ims{1} - ims{2};
elseif image2MinusImage1(ims,scalars)
Z = ims{2} - ims{1};
elseif imagePlusScalar(ims,scalars)
Z = ims{1} + scalars(2);
else
Z = images.internal.imlincombc(ims, scalars, outputClass);
end
else
Z = images.internal.imlincombc(ims, scalars, outputClass);
end
%%%%%%%%%%%%%%%%%%%%%%%%%%%%%%%%%%%%%%%%%%%%%%%%%%%%%%%%%%%%%%%%%%%%%%%%%%%%%%%%
function valid = imagePlusImage(images,scalars)
valid = numel(images) == 2 && numel(scalars) == 2 && ...
all(scalars == 1);
%%%%%%%%%%%%%%%%%%%%%%%%%%%%%%%%%%%%%%%%%%%%%%%%%%%%%%%%%%%%%%%%%%%%%%%%%%%%%%%%
function valid = image1MinusImage2(images,scalars)
valid = numel(images) == 2 && numel(scalars) == 2 && ...
scalars(1) == 1 && scalars(2) == -1;
%%%%%%%%%%%%%%%%%%%%%%%%%%%%%%%%%%%%%%%%%%%%%%%%%%%%%%%%%%%%%%%%%%%%%%%%%%%%%%%%
function valid = image2MinusImage1(images,scalars)
valid = numel(images) == 2 && numel(scalars) == 2 && ...
scalars(1) == -1 && scalars(2) == 1;
%%%%%%%%%%%%%%%%%%%%%%%%%%%%%%%%%%%%%%%%%%%%%%%%%%%%%%%%%%%%%%%%%%%%%%%%%%%%%%%%
function valid = imagePlusScalar(images,scalars)
valid = numel(images) == 1 && numel(scalars) == 2 && ...
scalars(1) == 1;
%%%%%%%%%%%%%%%%%%%%%%%%%%%%%%%%%%%%%%%%%%%%%%%%%%%%%%%%%%%%%%%%%%%%%%%%%%%%%%%%
function [images, scalars, output_class] = ParseInputs(varargin)
narginchk(2, Inf);
if ischar(varargin{end})
valid_strings = {'uint8' 'uint16' 'uint32' 'int8' 'int16' 'int32' ...
'single' 'double'};
output_class = validatestring(varargin{end}, valid_strings, mfilename, ...
'OUTPUT_CLASS', 3);
varargin(end) = [];
else
```

```
if islogical(varargin{2})
output_class = 'double';
else
output_class = class(varargin{2});
end
end
%check images
images = varargin(2:2:end);
if ~iscell(images) || isempty(images)
displayInternalError('images');
end
% assign and check scalars
for p = 1:2:length(varargin)
validateattributes(varargin{p}, {'double'}, {'real' 'nonsparse' 'scalar'},
...
mfilename, sprintf('K%d', (p+1)/2), p);
end
scalars = [varargin{1:2:end}];
%make sure it is a vector
if ( ~ismatrix(scalars) || (all(size(scalars)~=1) && any(size(scalars)~=0)) )
displayInternalError('scalars');
end
%%%%%%%%%%%%%%%%%%%%%%%%%%%%%%%%%%%%%%%%%%%%%%%%%%%%%%%%%%%%%%%%%%%%%%%%%%
function displayInternalError(string)
error(message('images:imlincomb:internalError', upper( string )))
```
